# 

**Published:** 1892-11

**Authors:** 


### CONTENTS.

PROCEEDINGS.

American Dental Association .... 517
The Colorado State Dental Association .... 541
Odontalgia Cured by Ipecac .... 542

COMMUNICATIONS.

What Changes occur in the Teeth with the Advancement of Age .... 543

SELECTIONS.

Gluten Foods .... 547
The Doctor as a Popular Leader .... 551
Reminders of the Past .... 552
Patent Medicines in Turkey .... 552
Ratio of Physicians to Population .... 552
Neuralgias and Neuralgic Affections .... 553
To Stimulate Appetite .... 554
The Natural Result .... 554
Walking .... 555
The Cure of Tetanus with the Antitoxin Obtained from the Serum of an Immune Animal .... 555
Facial Paralysis .... 556
The Keely Treatment .... 556
To Remove Rust .... 557
Boils .... 557
Therapeutics of Gold .... 558
Keep Your Head Clean .... 558
The Moral Side .... 559
Cocaine Poisoning .... 559
Salicylic Acid .... 560
Dental Neuralgia .... 560
A Method of Bleaching Sponges .... 561
Defining a Blush .... 561
Character .... 561
The True Physician .... 562
Simple Water Test .... 562

EDITORIAL.

The Columbian Dental Congress .... 563
West Virginia State Dental Society .... 563
Ohio State Dental Society .... 564
The Pan-American Medical Congress .... 565
Knoxville City Dental Society .... 568
Board of Examiners .... 568

